# 1,3-Dihydr­oxy-9,10-dioxo-9,10-di­hydro­anthracene-2-carbaldehyde

**DOI:** 10.1107/S1600536808004169

**Published:** 2008-02-15

**Authors:** Khalijah Awang, Nor Hadiani Ismail, Rohaya Ahmad, Nor Hafizoh Saidan, Pascal Retailleau

**Affiliations:** a Chemistry Department, Faculty of Science, University of Malaya, 50603 Kuala Lumpur, Malaysia; b Faculty of Applied Sciences, Universiti Teknologi MARA Malaysia, 40450 Shah Alam, Selangor, Malaysia; c ICSN-CNRS, 1 avenue de la Terrasse, 91198 Gif sur Yvette, France

## Abstract

The title compound, C_15_H_8_O_5_, also known as nordamnacanthal, was isolated from the Malaysian *Morinda citrifolia* L. The 20 non-H atoms are coplanar. The structure is stabilized by intra­molecular O—H⋯O hydrogen bonds and inter­molecular O—H⋯O and C—H⋯O hydrogen bonds, forming bilayers of mol­ecular tapes with alternating stacking directions along the *a* axis.

## Related literature

For related literature, see: Chan-Blanco *et al.* (2006 ▶); Ismail (1998 ▶); Ohsawa & Ohba (1993 ▶); Singh *et al.* (1984 ▶); Whistler (1985 ▶); Wijnsma & Verpoorte (1986 ▶); Zhu *et al.* (2008 ▶).
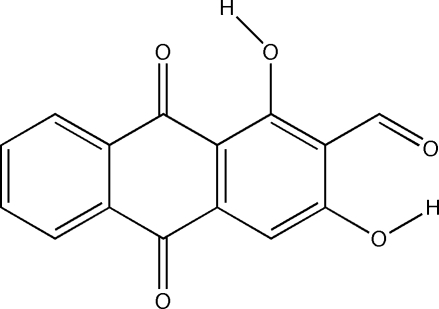


## Experimental

### 

#### Crystal data

C_15_H_8_O_5_
*M* *_r_* = 268.21
Monoclinic, 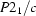

*a* = 10.547 (2) Å
*b* = 5.669 (1) Å
*c* = 20.231 (3) Å
β = 110.62 (4)°
*V* = 1132.1 (5) Å^3^
*Z* = 4
Mo *K*α radiation
μ = 0.12 mm^−1^
*T* = 293 (2) K
0.60 × 0.39 × 0.14 mm

#### Data collection

Nonius KappaCCD diffractometer
Absorption correction: none
14030 measured reflections
2298 independent reflections
1554 reflections with *I* > 2σ(*I*)
*R*_int_ = 0.029

#### Refinement

*R*[*F* ^2^ > 2σ(*F* ^2^)] = 0.051
*wR*(*F* ^2^) = 0.150
*S* = 1.06
2296 reflections
181 parameters
H-atom parameters constrained
Δρ_max_ = 0.27 e Å^−3^
Δρ_min_ = −0.20 e Å^−3^

### 

Data collection: *DENZO* (Otwinowski & Minor, 1997 ▶) and *COLLECT* (Nonius, 1999 ▶); cell refinement: *DENZO* and *COLLECT*; data reduction: *SCALEPACK* (Otwinowski & Minor, 1997 ▶); program(s) used to solve structure: *SIR97* (Altomare *et al.*, 1999 ▶); program(s) used to refine structure: *SHELXL97* (Sheldrick, 2008 ▶); molecular graphics: *PLATON* (Spek, 2003 ▶) and *Mercury* (Macrae *et al.*, 2006 ▶); software used to prepare material for publication: *SHELXL97* and *publCIF* (Westrip, 2008 ▶).

## Supplementary Material

Crystal structure: contains datablocks I, global. DOI: 10.1107/S1600536808004169/bg2162sup1.cif

Structure factors: contains datablocks I. DOI: 10.1107/S1600536808004169/bg2162Isup2.hkl

Additional supplementary materials:  crystallographic information; 3D view; checkCIF report

## Figures and Tables

**Table 1:** Hydrogen-bond geometry (Å, °)

*D*—H⋯*A*	*D*—H	H⋯*A*	*D*⋯*A*	*D*—H⋯*A*
O3—H3⋯O2	0.82	1.86	2.590 (3)	148
O1—H1⋯O5	0.82	1.86	2.577 (2)	146
O1—H1⋯O5^i^	0.82	2.34	2.933 (2)	130
C4—H4⋯O4^ii^	0.93	2.45	3.358 (2)	166
C10—H10⋯O2^iii^	0.93	2.53	3.312 (3)	142

Symmetry codes: (i) 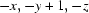; (ii) 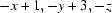; (iii) 
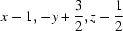
.

**Fig. 1.**
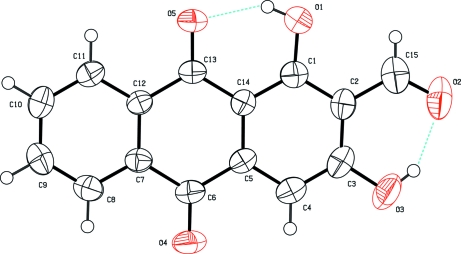
Perspective view of the title compound with the atom numbering; displacement ellipsoids are at the 50% probability level. The intramolecular O—H···O interactions are shown as dotted lines.

**Fig. 2.**
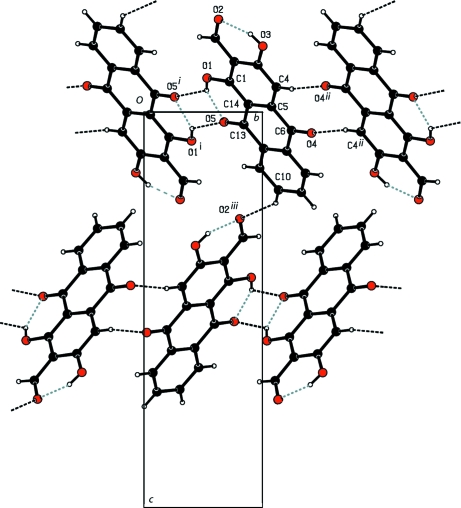
Part of the crystal structure showing non-parallel molecular slabs forming herringbone pattern along a. (Intra-)Inter-molecular hydrogen bonds are indicated by (dotted) dashed lines. Symmetry codes: as in Table 1.

**Fig. 3.**
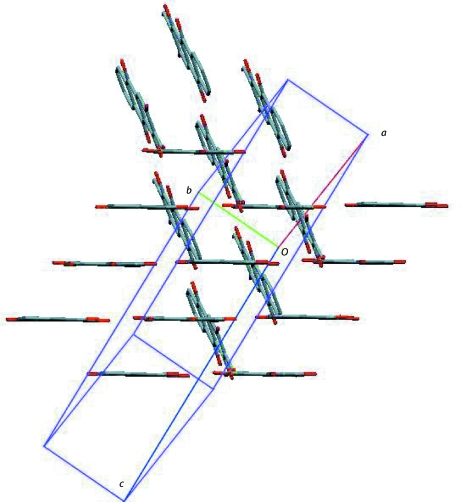
The crystal packing showing the two orientations taken by the stacking of molecular tapes. Note the offset between successive layers.

## supplementary crystallographic information

### Comment

*Morinda citrifolia* Linn. (Noni), has been one of the most used traditional folk medicinal plants in Polynesia for over 2000 years (Whistler,1985). It has been reported to have a broad range of therapeutic and nutritional properties (Chan-Blanco *et al.*,2006) including antibacterial, antiviral, antifungal, antitumor, analgesic, hypotensive, anti-inflammatory and immune enhancing effects (Singh *et al.*, 1984). Nordamnacanthal, damnacanthal and morindone (Ismail, 1998; Wijnsma & Verpoorte, 1986) have been isolated from the Malaysian *Morinda citrifolia* Linn. The crystal structure of damnacanthal having been reported by Ohsawa & Ohba (1993), we present in this communication the crystal structure of nordamnacanthal (I). (Fig. 1) shows its molecular structure. The C—C bond lengths in the anthraquinone ring range from 1.377 (3) Å to 1.484 (3) Å, the carbonyl bond distances from 1.220 (2) Å to 1.237 (2) Å and the two hydroxyl bond distances are 1.326 (2) Å and 1.349 (2) Å; all are comparable to those observed in similar structures (Ohsawa & Ohba, 1993; Zhu *et al.*, 2008). All 20 non-H atoms of (I) are essentially coplanar, their mean deviation from the least-squares molecular plane being 0.028 Å and the dihedral angle between the two benzene rings being 1.27 (10)°. The molecule features two intramolecular O—H···O hydrogen bonds, with O3···O2 distance of 2.590 (3) Å and O1···O5 distance of 2.577 (2) Å. Additionally, atom O1 is also engaged into an intermolecular hydrogen bond with atom O5, *viz*. O1—H1···O5^i^ [symmetry code:(i) -*x*, 1 - *y*, -*z*] leading to the formation of a coplanar centrosymmetric dimer *via* the key {H—O1—C1—C14—C13—O5}2 synthon, *R*_2_^2^(12). Adjacent dimers extend through synthon *R*_2_^2^(10) of weak C4—H4···O4^ii^ [symmetry code:(ii) 1 - *x*, 3 - *y*, -*z*] hydrogen bond to form molecular tapes running parallel to the [120] and [120] directions (Fig. 2). The dihedral angle between the two molecular tape orientations is 66.03° and an additional weak C10—H10···O4^iii^ [symmetry code:(ii) -1 + *x*, 3/2 - *y*, -1/2 + *z*] hydrogen bond links the tapes along the *c* axis. The tapes are stacked along the *a* axis, forming two kinds of layers in which molecules related by an inversion center stack with an interplanar spacing of 3.255 (4) Å and a centroid offset of *ca* 3.5 Å (Fig. 3).

### Experimental

*Morinda citrifolia* used in this study was collected from kg. Tanjung Keramat, Langkap, Perak. The roots were harvested, washed, chopped into small pieces and then dried at room temperature for one week. The dried sample was then ground to small size using grinder. The ground roots (1.5 kg) were soaked at room temperature in dichloromethane for 48 h. The solvent was then removed by filtration and fresh solvent added to the plant material. The extraction was repeated three times. The combined filtrate was evaporated under reduced pressure to give brown coloured residue (35.6 g). The crude extract was fractionated using Medium Pressure Liquid Chromatography (MPLC) system fitted with Buchi Pump Module C-601. The sample (10 g) was introduced dry after being pre-absorbed onto acid-washed silica gel (10 g) in two portions. The column (150 mm *x* 40 mm) was packed with 90 g acid-washed silica gel (Merck 7734) and eluted gradiently with petroleum ether, chloroform and chloroform enriched with increasing percentages of methanol (1%, 2% and 5%). Seven combined fractions were collected based on thin layer chromatography (TLC) pattern (labeled A, B, C, D, E, F, and G).

Nordamnacanthal (1.65 g) were isolated from fraction A after column chromatography. The fraction was re-chromatographed using small column (400 mm *x* 20 mm) packed with 2% acid-washed silica gel (Merck 9385) eluted gradiently with petroleum ether and chloroform. The first orange band eluted out from the column was collected in small vials and inspected using analytical TLC developed in PE:CHCl3 (4:6) showing a single spot of a purified compound. Recrystallization from hot CHCl3 gave bright orange crystals.

### Refinement

All H atoms were located in difference maps but then were treated as riding in geometrically positions, with O—H = 0.82 Å, and C—H = 0.93 Å (sp2) and with *U*_iso_(H) = 1.2*U*_eq_(carrier).

### Crystal data

**Table:**

C_15_H_8_O_5_	*F*_000_ = 552
*M**_r_* = 268.21	*D*_x_ = 1.574 Mg m^−^^3^
Monoclinic, *P*2_1_/*c*	Mo *K*α radiation λ = 0.71070 Å
Hall symbol: -P 2ybc	Cell parameters from 10451 reflections
*a* = 10.547 (2) Å	θ = 0.4–26.4º
*b* = 5.669 (1) Å	µ = 0.12 mm^−^^1^
*c* = 20.231 (3) Å	*T* = 293 (2) K
β = 110.62 (4)º	Prism, orange
*V* = 1132.1 (5) Å^3^	0.60 × 0.39 × 0.14 mm
*Z* = 4	

### Data collection

**Table:**

Nonius KappaCCD diffractometer	1554 reflections with *I* > 2σ(*I*)
Radiation source: fine-focus sealed tube	*R*_int_ = 0.029
Monochromator: graphite	θ_max_ = 26.4º
*T* = 293(2) K	θ_min_ = 2.1º
φ and ω scans	*h* = 0→13
Absorption correction: none	*k* = −7→0
14030 measured reflections	*l* = −25→22
2298 independent reflections	

### Refinement

**Table:**

Refinement on *F*^2^	Secondary atom site location: difference Fourier map
Least-squares matrix: full	Hydrogen site location: difference Fourier map
*R*[*F*^2^ > 2σ(*F*^2^)] = 0.051	H-atom parameters constrained
*wR*(*F*^2^) = 0.150	*w* = 1/[σ^2^(*F*_o_^2^) + (0.0705*P*)^2^ + 0.3487*P*] where *P* = (*F*_o_^2^ + 2*F*_c_^2^)/3
*S* = 1.06	(Δ/σ)_max_ < 0.001
2296 reflections	Δρ_max_ = 0.27 e Å^−^^3^
181 parameters	Δρ_min_ = −0.20 e Å^−^^3^
Primary atom site location: structure-invariant direct methods	Extinction correction: none

### Special details

**Table:**

Geometry. All e.s.d.'s (except the e.s.d. in the dihedral angle between two l.s. planes) are estimated using the full covariance matrix. The cell e.s.d.'s are taken into account individually in the estimation of e.s.d.'s in distances, angles and torsion angles; correlations between e.s.d.'s in cell parameters are only used when they are defined by crystal symmetry. An approximate (isotropic) treatment of cell e.s.d.'s is used for estimating e.s.d.'s involving l.s. planes.
Refinement. Refinement of *F*^2^ against ALL reflections (2298) except two reflections with Delta(*F*^2^)/e.s.d. greater than 9 (2296). The weighted *R*-factor *wR* and goodness of fit *S* are based on *F*^2^, conventional *R*-factors *R* are based on *F*, with *F* set to zero for negative *F*^2^. The threshold expression of *F*^2^ > σ(*F*^2^) is used only for calculating *R*-factors(gt) *etc*. and is not relevant to the choice of reflections for refinement. *R*-factors based on *F*^2^ are statistically about twice as large as those based on *F*, and *R*- factors based on ALL data will be even larger.

### Fractional atomic coordinates and isotropic or equivalent isotropic displacement parameters (Å^2^)

**Table:**

	*x*	*y*	*z*	*U*_iso_*/*U*_eq_	
O4	0.32777 (16)	1.4401 (3)	−0.05914 (8)	0.0669 (5)	
O5	0.02849 (14)	0.7077 (2)	−0.03142 (7)	0.0531 (4)	
O1	0.22406 (15)	0.5649 (2)	0.07880 (7)	0.0525 (4)	
H1	0.1487	0.5610	0.0479	0.063*	
O3	0.62014 (15)	1.0321 (3)	0.15870 (9)	0.0746 (5)	
H3	0.6408	0.9283	0.1889	0.090*	
O2	0.58762 (18)	0.6610 (3)	0.22568 (9)	0.0811 (6)	
C6	0.2604 (2)	1.2727 (3)	−0.05264 (10)	0.0437 (5)	
C7	0.12238 (19)	1.2323 (3)	−0.10446 (9)	0.0388 (5)	
C12	0.04523 (18)	1.0390 (3)	−0.09796 (9)	0.0364 (4)	
C13	0.09986 (19)	0.8728 (3)	−0.03790 (10)	0.0388 (5)	
C14	0.23549 (18)	0.9106 (3)	0.01290 (10)	0.0377 (4)	
C5	0.31419 (18)	1.1053 (3)	0.00715 (10)	0.0396 (5)	
C4	0.4429 (2)	1.1451 (4)	0.05601 (11)	0.0493 (5)	
H4	0.4935	1.2742	0.0513	0.059*	
C3	0.4949 (2)	0.9898 (4)	0.11180 (11)	0.0521 (6)	
C2	0.4211 (2)	0.7925 (4)	0.11979 (10)	0.0461 (5)	
C1	0.2905 (2)	0.7533 (3)	0.06963 (10)	0.0425 (5)	
C15	0.4762 (3)	0.6333 (4)	0.17886 (13)	0.0652 (7)	
H15	0.4247	0.5031	0.1817	0.078*	
C11	−0.0846 (2)	1.0056 (4)	−0.14710 (10)	0.0446 (5)	
H11	−0.1359	0.8766	−0.1430	0.054*	
C10	−0.1371 (2)	1.1638 (4)	−0.20178 (10)	0.0509 (5)	
H10	−0.2239	1.1414	−0.2346	0.061*	
C9	−0.0613 (2)	1.3547 (4)	−0.20791 (11)	0.0539 (6)	
H9	−0.0975	1.4613	−0.2447	0.065*	
C8	0.0679 (2)	1.3894 (4)	−0.15996 (10)	0.0486 (5)	
H8	0.1185	1.5183	−0.1648	0.058*	

### Atomic displacement parameters (Å^2^)

**Table:**

	*U*^11^	*U*^22^	*U*^33^	*U*^12^	*U*^13^	*U*^23^
O4	0.0621 (10)	0.0626 (10)	0.0708 (11)	−0.0288 (8)	0.0169 (8)	0.0066 (8)
O5	0.0512 (9)	0.0466 (8)	0.0591 (9)	−0.0150 (7)	0.0163 (7)	0.0068 (7)
O1	0.0511 (9)	0.0471 (8)	0.0565 (9)	−0.0020 (7)	0.0155 (7)	0.0087 (7)
O3	0.0437 (9)	0.0866 (12)	0.0726 (11)	−0.0075 (8)	−0.0056 (8)	−0.0060 (9)
O2	0.0657 (11)	0.0934 (13)	0.0606 (11)	0.0183 (10)	−0.0071 (9)	0.0054 (9)
C6	0.0458 (11)	0.0411 (11)	0.0474 (11)	−0.0107 (9)	0.0201 (9)	−0.0052 (9)
C7	0.0434 (11)	0.0367 (10)	0.0381 (10)	−0.0049 (8)	0.0167 (9)	−0.0044 (8)
C12	0.0367 (10)	0.0360 (10)	0.0374 (10)	−0.0028 (8)	0.0142 (8)	−0.0048 (8)
C13	0.0419 (11)	0.0355 (10)	0.0421 (11)	−0.0054 (8)	0.0187 (9)	−0.0036 (8)
C14	0.0370 (10)	0.0388 (10)	0.0378 (10)	0.0018 (8)	0.0138 (8)	−0.0027 (8)
C5	0.0359 (10)	0.0413 (10)	0.0426 (11)	−0.0048 (8)	0.0152 (8)	−0.0075 (8)
C4	0.0450 (12)	0.0496 (12)	0.0536 (13)	−0.0085 (9)	0.0176 (10)	−0.0075 (10)
C3	0.0386 (11)	0.0605 (13)	0.0511 (13)	0.0012 (10)	0.0081 (10)	−0.0113 (11)
C2	0.0427 (11)	0.0504 (12)	0.0423 (11)	0.0075 (9)	0.0112 (9)	−0.0026 (9)
C1	0.0432 (11)	0.0404 (11)	0.0474 (11)	0.0016 (8)	0.0202 (9)	−0.0033 (8)
C15	0.0664 (15)	0.0655 (15)	0.0561 (14)	0.0136 (12)	0.0122 (12)	0.0028 (12)
C11	0.0413 (11)	0.0467 (11)	0.0457 (11)	−0.0068 (9)	0.0149 (9)	−0.0065 (9)
C10	0.0433 (11)	0.0603 (13)	0.0430 (11)	0.0026 (10)	0.0075 (9)	−0.0059 (10)
C9	0.0634 (14)	0.0528 (13)	0.0422 (12)	0.0073 (11)	0.0146 (11)	0.0072 (9)
C8	0.0578 (13)	0.0430 (11)	0.0471 (12)	−0.0066 (9)	0.0209 (11)	0.0014 (9)

### Geometric parameters (Å, °)

**Table:**

O4—C6	1.220 (2)	C14—C5	1.410 (3)
O5—C13	1.237 (2)	C5—C4	1.388 (3)
O1—C1	1.326 (2)	C4—C3	1.383 (3)
O1—H1	0.8200	C4—H4	0.9300
O3—C3	1.349 (3)	C3—C2	1.404 (3)
O3—H3	0.8200	C2—C1	1.411 (3)
O2—C15	1.233 (3)	C2—C15	1.446 (3)
C6—C7	1.482 (3)	C15—H15	0.9300
C6—C5	1.484 (3)	C11—C10	1.380 (3)
C7—C8	1.389 (3)	C11—H11	0.9300
C7—C12	1.399 (2)	C10—C9	1.377 (3)
C12—C11	1.393 (3)	C10—H10	0.9300
C12—C13	1.484 (3)	C9—C8	1.380 (3)
C13—C14	1.454 (3)	C9—H9	0.9300
C14—C1	1.407 (3)	C8—H8	0.9300
			
C1—O1—H1	109.5	O3—C3—C2	120.4 (2)
C3—O3—H3	109.5	C4—C3—C2	121.61 (18)
O4—C6—C7	120.56 (18)	C3—C2—C1	118.88 (18)
O4—C6—C5	121.01 (18)	C3—C2—C15	121.0 (2)
C7—C6—C5	118.43 (16)	C1—C2—C15	120.1 (2)
C8—C7—C12	119.32 (18)	O1—C1—C14	122.54 (18)
C8—C7—C6	119.77 (17)	O1—C1—C2	117.17 (18)
C12—C7—C6	120.91 (17)	C14—C1—C2	120.29 (18)
C11—C12—C7	119.84 (17)	O2—C15—C2	123.5 (3)
C11—C12—C13	119.92 (16)	O2—C15—H15	118.2
C7—C12—C13	120.23 (16)	C2—C15—H15	118.2
O5—C13—C14	121.38 (17)	C10—C11—C12	120.01 (19)
O5—C13—C12	119.46 (17)	C10—C11—H11	120.0
C14—C13—C12	119.15 (16)	C12—C11—H11	120.0
C1—C14—C5	118.53 (17)	C9—C10—C11	120.08 (19)
C1—C14—C13	120.26 (17)	C9—C10—H10	120.0
C5—C14—C13	121.21 (17)	C11—C10—H10	120.0
C4—C5—C14	121.66 (18)	C10—C9—C8	120.6 (2)
C4—C5—C6	118.30 (17)	C10—C9—H9	119.7
C14—C5—C6	120.04 (17)	C8—C9—H9	119.7
C3—C4—C5	119.03 (19)	C9—C8—C7	120.12 (19)
C3—C4—H4	120.5	C9—C8—H8	119.9
C5—C4—H4	120.5	C7—C8—H8	119.9
O3—C3—C4	118.0 (2)		
			
O4—C6—C7—C8	−1.6 (3)	C6—C5—C4—C3	−179.58 (18)
C5—C6—C7—C8	178.31 (17)	C5—C4—C3—O3	−179.91 (17)
O4—C6—C7—C12	179.08 (18)	C5—C4—C3—C2	0.5 (3)
C5—C6—C7—C12	−1.0 (3)	O3—C3—C2—C1	−179.82 (18)
C8—C7—C12—C11	0.3 (3)	C4—C3—C2—C1	−0.3 (3)
C6—C7—C12—C11	179.58 (17)	O3—C3—C2—C15	1.2 (3)
C8—C7—C12—C13	−178.19 (17)	C4—C3—C2—C15	−179.25 (19)
C6—C7—C12—C13	1.1 (3)	C5—C14—C1—O1	−179.51 (17)
C11—C12—C13—O5	−1.1 (3)	C13—C14—C1—O1	1.1 (3)
C7—C12—C13—O5	177.34 (17)	C5—C14—C1—C2	0.7 (3)
C11—C12—C13—C14	−179.93 (16)	C13—C14—C1—C2	−178.70 (17)
C7—C12—C13—C14	−1.5 (3)	C3—C2—C1—O1	179.83 (17)
O5—C13—C14—C1	2.3 (3)	C15—C2—C1—O1	−1.2 (3)
C12—C13—C14—C1	−178.89 (16)	C3—C2—C1—C14	−0.3 (3)
O5—C13—C14—C5	−177.04 (18)	C15—C2—C1—C14	178.65 (18)
C12—C13—C14—C5	1.8 (3)	C3—C2—C15—O2	1.2 (4)
C1—C14—C5—C4	−0.4 (3)	C1—C2—C15—O2	−177.7 (2)
C13—C14—C5—C4	178.95 (17)	C7—C12—C11—C10	−0.4 (3)
C1—C14—C5—C6	178.98 (16)	C13—C12—C11—C10	178.07 (17)
C13—C14—C5—C6	−1.7 (3)	C12—C11—C10—C9	0.0 (3)
O4—C6—C5—C4	0.6 (3)	C11—C10—C9—C8	0.4 (3)
C7—C6—C5—C4	−179.34 (17)	C10—C9—C8—C7	−0.5 (3)
O4—C6—C5—C14	−178.84 (19)	C12—C7—C8—C9	0.2 (3)
C7—C6—C5—C14	1.3 (3)	C6—C7—C8—C9	−179.14 (18)
C14—C5—C4—C3	−0.2 (3)		

### Hydrogen-bond geometry (Å, °)

**Table:**

*D*—H···*A*	*D*—H	H···*A*	*D*···*A*	*D*—H···*A*
O3—H3···O2	0.82	1.86	2.590 (3)	148
O1—H1···O5	0.82	1.86	2.577 (2)	146
O1—H1···O5^i^	0.82	2.34	2.933 (2)	130
C4—H4···O4^ii^	0.93	2.45	3.358 (2)	166
C10—H10···O2^iii^	0.93	2.53	3.312 (3)	142

Symmetry codes: (i) −*x*, −*y*+1, −*z*; (ii) −*x*+1, −*y*+3, −*z*; (iii) *x*−1, −*y*+3/2, *z*−1/2.
